# Case Report: The “metal ring” phenomenon: a rare cause of recurrent in-stent restenosis

**DOI:** 10.3389/fcvm.2026.1869115

**Published:** 2026-06-25

**Authors:** Xuefei Mu, Ziqi Li, Bin Wang, Kai Xu, Dali Zhang, Quanmin Jing, Yaling Han

**Affiliations:** Laboratory of Frigid Zone Cardiovascular Disease, Department of Cardiology, General Hospital of the PLA Northern Theater Command, Shenyang, Liaoning, China

**Keywords:** coronary stent fracture, in-stent restenosis, metal ring, modified Side Flap Technique, optical coherence tomography

## Abstract

**Background:**

Recurrent in-stent restenosis (ISR) remains a challenging issue in coronary intervention. Localized intractable ISR caused by a non-expandable metal ring is extremely rare. This report describes a rare case of recurrent in-stent restenosis attributable to a non-dilatable metal ring and demonstrates a viable interventional strategy for its management.

**Case presentation:**

A 64-year-old female patient was admitted with complaints of “intermittent chest pain for over one year, worsening over the past 10 days”. In June 2022, the patient underwent drug-eluting stent implantation in the proximal-to-mid right coronary artery (RCA) for acute coronary syndrome. She experienced recurrent angina at 4 and 9 months post-procedure, with coronary angiography on both occasions revealing 95% ISR in the RCA. During the third intervention on January 31, 2023, optical coherence tomography (OCT) identified a non-dilatable metal ring at the site of severe stenosis. Attempts to disrupt the ring using intravascular lithotripsy and high-pressure non-compliant balloon inflation were unsuccessful. Ultimately, a modified Side Flap Technique was employed: a guidewire was advanced through the lesion lateral to the metal ring, enabling successful stent-in-stent implantation at the original site of the metal ring in the mid-RCA with adequate expansion. Post-procedural symptoms resolved substantially, and the patient remained free of major adverse cardiovascular events during three years of follow-up.

**Conclusion:**

Non-dilatable intracoronary metal rings represent an exceedingly rare cause of recurrent ISR following stent implantation. OCT plays an essential role in identifying such structural lesions. When conventional dilation techniques fail, a modified Side Flap Technique combined with stent-in-stent strategy may represent a feasible bail-out approach in this clinical setting.

## 1 Introduction

With the widespread application of drug-eluting stents (DES), the incidence of Coronary Stent Fracture (CSF) has decreased significantly; however, the majority of literature reports a CSF incidence ranging from 0.84% to 5.8% (1–3). To heighten awareness regarding this persistent clinical challenge, Nature Reviews Cardiology specifically published a review article dedicated to CSF in 2019 (4). Various clinical classification systems have been established to characterize this condition, such as the Popma classification based on angiographic morphology and the Doi classification relying on intravascular ultrasound (IVUS) imaging (5, 6).

Currently, the main methods used to evaluate coronary stents in clinical practice include Coronary Angiography (CAG), IVUS, Optical Coherence Tomography (OCT), Multi-detector Computed Tomography (MDCT), and StentBoost (7, 8); however, mechanical obstruction caused by non-dilatable metal rings is exceptionally rare. Failure to identify these structures preoperatively may lead to repeated interventional failures and poor clinical outcomes.

Intracoronary imaging is pivotal for assessing CSF and optimizing interventions. OCT is the gold standard for visualizing stent morphology and identifying mechanisms like fracture, underexpansion, and neoatherosclerosis (9, 10). For mechanical obstructions caused by non-dilatable metal rings—often resistant to conventional balloon dilation—therapeutic options include atherectomy strategies (rotational, orbital, or Excimer Laser Coronary Atherectomy). Furthermore, while the conventional Side Flap Technique (11) has been described for wiring through crushed stent layers, we applied a modified Side Flap Technique tailored for this specific scenario14. Nevertheless, managing recurrent in-stent restenosis (ISR) associated with stent fracture remains challenging, frequently requiring individualized approaches guided by intracoronary imaging.

This case report describes a patient with localized intractable ISR characterized by early recurrence after DES implantation and resistance to repeated balloon angioplasty. OCT clearly revealed the etiology as a coiled fractured stent segment, manifesting as a non-dilatable “metal ring,” a mechanism that has been sparsely documented in the literature. Successful revascularization was achieved using a modified Side Flap Technique combined with stent-in-stent implantation, providing an important clinical reference for the recognition and management of this rare complication.

## 2 Case presentation

### 2.1 Patient demographics

A 64-year-old female presented with intermittent chest pain for over one year, worsening over the past 10 days. Her cardiovascular risk profile included well-controlled hypertension (approximately 120/80 mmHg), type 2 diabetes mellitus (fasting blood glucose: 6–8 mmol/L, 2-hour postprandial glucose: 9–10 mmol/L), and dyslipidemia (LDL-C: 1.2–1.64 mmol/L on statins). Renal function was normal. The patient had no history of smoking or alcohol consumption. Her antiplatelet regimen consisted of aspirin (100 mg daily) and ticagrelor (90 mg twice daily), with good compliance.

**Figure 1:**
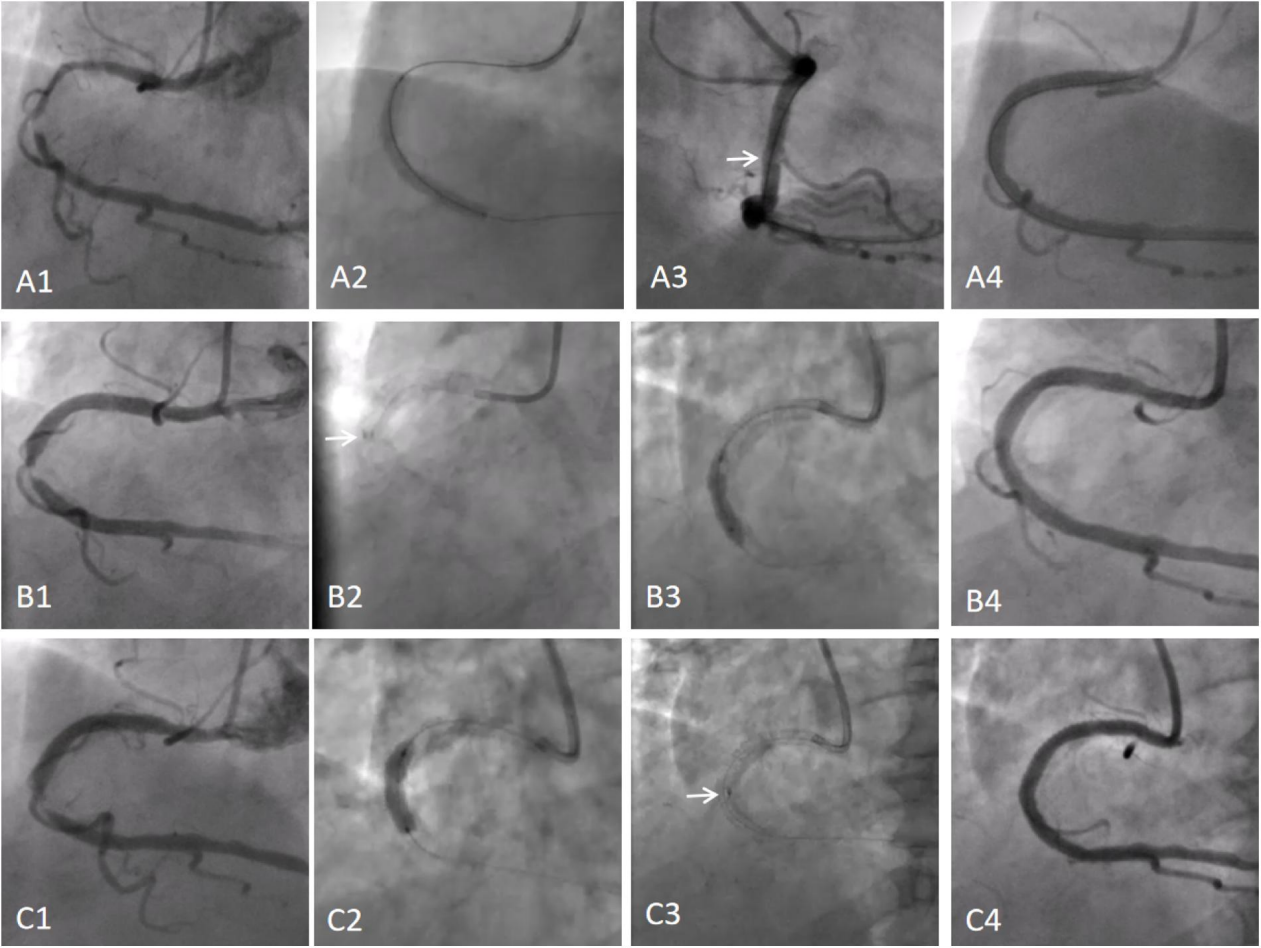
Coronary angiographic timeline of three interventions in the right coronary artery. **(A)** Initial PCI: A1, baseline CAG (left anterior oblique view) showing severe stenosis in the proximal-to-mid right coronary artery; A2, intraprocedural high-pressure post-dilation with a non-compliant balloon; A3, stent strut fracture (arrow) with plaque protrusion (white) and local contrast filling defect; A4, final angiographic result after the index procedure, demonstrating a patent vessel without an obvious metal ring. **(B)** First ISR intervention: B1, pre-procedural angiography showing significant ISR with marked luminal narrowing; B2, the metal ring (two radiopaque dots; arrow); B3, balloon inflation dilating the stenotic segment; B4, post-procedural angiographic result with improved luminal gain. **(C)** Final intervention using the modified Side Flap Technique: C1, pre-procedural angiography; C2, intraprocedural deployment of a new stent; C3, complete apposition of the metal ring (two radiopaque dots merged into one; arrow); C4, final post-procedural angiography demonstrating restored vessel patency.

On June 6, 2022, the patient underwent index percutaneous coronary intervention (PCI), during which two DES (3.5 × 36 mm and 3.5 × 33 mm) were sequentially deployed in the right coronary artery (RCA). However, fluoroscopic imaging in the right anterior oblique view revealed a circular radiopacity located distal to the right marginal branch (Figure 1). This finding was overlooked on initial angiographic assessment; retrospective review confirmed it represented the earliest angiographic sign of stent fracture: the fractured stent segment gradually recoiled and coiled, forming the complete metal ring that was identified in subsequent interventions. Following the procedure, the patient's symptoms improved, and she was discharged on guideline-directed dual antiplatelet therapy.

### 2.2 First restenosis episode

Four months later (October 18, 2022), the patient presented with recurrent angina. Angiography revealed 95% ISR in the mid-RCA, along with two small bright spots within the stent (Figure 1). IVUS confirmed stent fracture, evidenced by discontinuous struts and a coiled circular configuration. Although an extension catheter had been used previously for the tortuous and calcified anatomy, it was withdrawn completely without any evidence of kinking, fracture, coating delamination, or tip detachment, thereby excluding retained catheter fragments or procedural complications. During the intervention, despite the absence of a waist sign during 3.5 mm non-compliant balloon inflation (suggesting adequate expansion), IVUS showed a severely restricted minimum stent area of 3.3 mm^2^. Consequently, the procedure was terminated.

### 2.3 Recalcitrant stenosis and differential diagnosis

On January 29, 2023, the patient presented with recurrent angina evidenced by two metallic high-density shadows in multiple projections (Figure 1). This rapid three-month recurrence, characterized by adequate balloon expansion but insufficient lumen gain and elastic recoil, suggested the presence of mechanically rigid metal rings resistant to conventional dilation. Multidisciplinary discussion attributed this non-dilatable lesion to severe calcification, leading to a recommendation for intravascular lithotripsy (IVL). Consequently, a third intervention was performed by a senior operator on January 31, 2023.

### 2.4 Bail-out intervention

**Figure 2:**
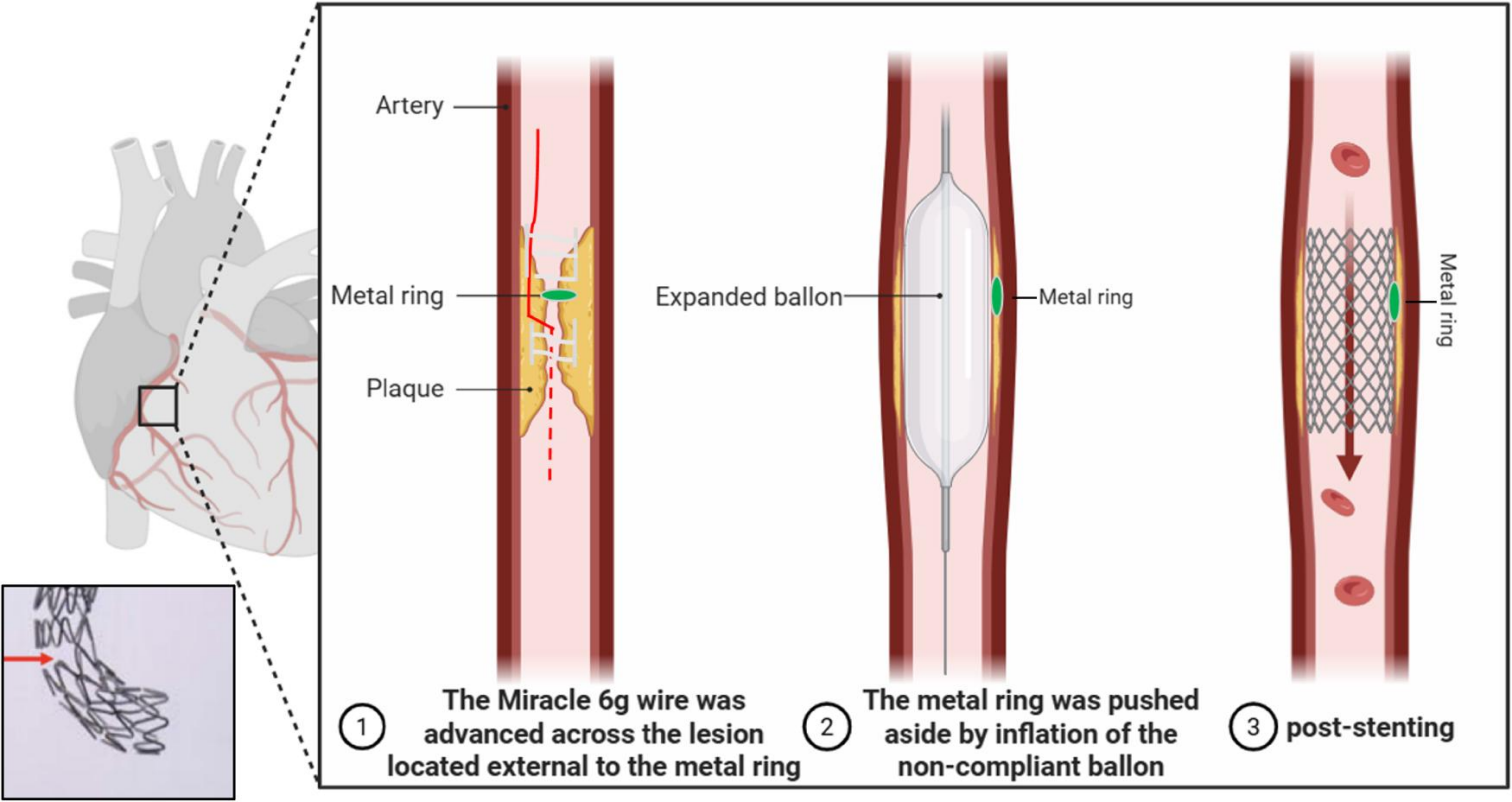
Schematic illustration of the modified Side Flap Technique. **(1)** The Miracle 6.0 guidewire was advanced through the lateral space outside the metal ring; **(2)** displacing the metal ring to the vessel wall by inflating a non-compliant balloon; **(3)** final result after stent-in-stent implantation restoring luminal patency. The inset illustrates the mechanism of stent strut fracture in tortuous vessels due to open-cell design.

**Figure 3:**
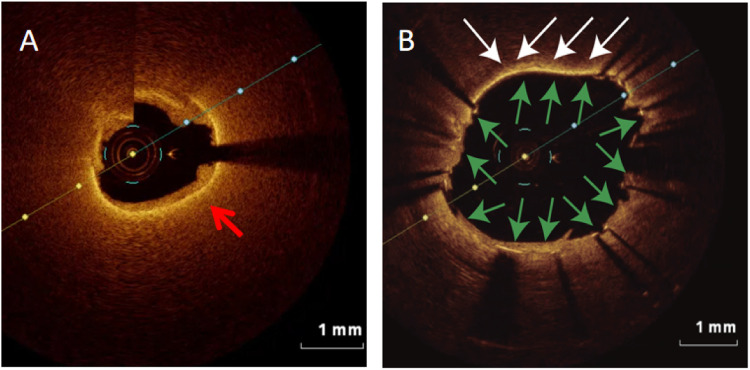
Optical coherence tomography (OCT) images demonstrating the modified Side Flap Technique. **(A)** The guidewire was advanced through the stent fracture site into the distal true lumen (red arrow). **(B)** After implantation of a new drug-eluting stent, the metal ring was compressed and fully apposed to the vessel wall (white arrows), with restoration of central lumen patency; green arrows indicate the struts of the newly implanted drug-eluting stent.

During the third procedure, a 6F JL3.5 guiding catheter was utilized to access the coronary artery. Lesion dilation was initially performed using a non-compliant balloon. Subsequent OCT examination revealed a rigid, complete metal ring with uniform thickness and a continuous circular configuration, distinct from intact stent struts; this structure demonstrated poor responsiveness to balloon dilation. This finding confirmed the presence of a fractured, retracted, and coiled stent segment, consistent with previous observations. Subsequently, IVL was administered to the mid-RCA under a standard protocol (6 ATM/cycle, 80 pulses); however, follow-up OCT indicated that the metal ring remained unchanged despite high-pressure balloon dilation and lithotripsy, with persistent luminal restriction. To address this challenge, a modified Side Flap Technique was employed. A Corsair microcatheter was utilized to facilitate the navigation of a Miracle 6 guidewire through the potential space between the metal ring and the vessel wall to access the distal true lumen, followed by exchange for a workhorse guidewire. A stent was then implanted to laterally displace and affix the metal ring against the vessel wall (Figure 2). Final angiography and OCT confirmed satisfactory stent expansion and apposition, demonstrating significant local lumen improvement with the metal ring effectively compressed against the vessel wall (Figure 3).

### 2.5 Follow-up results

Post-procedure chest pain resolved significantly without complications. The patient was discharged on intensified dual antiplatelet therapy and risk factor management. Major adverse cardiac events (MACE) were predefined as a composite of all-cause death, myocardial infarction, target lesion revascularization, stent thrombosis, and stroke. The follow-up protocol mandated outpatient visits at 1, 3, 6, and 12 months post-discharge, followed by annual assessments including symptom evaluation, ECG, enzyme assays, and echocardiography; exercise stress testing was scheduled at 6 months and 3 years. At the 3-year follow-up, the patient remained asymptomatic with no evidence of angina recurrence, TLR, MI, stent thrombosis, or bleeding events, and exercise stress tests revealed no ischemia. Given the absence of symptoms and a preference for non-invasive surveillance, repeat CAG or OCT was not performed. The clinical presentation and management timeline is summarized in Table 1.

**Table 1:** Clinical presentation and management timeline.

Date	Procedure	Key details
Baseline (June 6, 2022)	Index PCI	Implantation of 2 DES (3.5 × 36 mm, 3.5 × 33 mm) in RCA
Month 4 (October 18, 2022)	First ISR	Angiography showed restenosis; Following treatment with a cutting balloon and non-compliant balloon, IVUS revealed a metal ring and a minimal stent area (MSA) of only 3.3 mm^2^
Month 9 (January 29, 2023)	Second Recurrence	Angiography showed restenosis; OCT imaging revealed a metal ring. Despite attempts with high-pressure non-compliant balloons and IVL, treatment failed. Successful reconstruction of the lumen using the modified Side Flap Technique
Follow-up (3 years)	Follow-up	Clinical follow-up showed absence of recurrent angina, target lesion revascularization, myocardial infarction, stent thrombosis, or bleeding events

## 3 Discussion

We report a rare case of localized, intractable ISR secondary to a non-expandable “metal ring” resulting from stent fracture, a unique mechanical obstruction resistant to conventional interventions such as high-pressure balloon dilation and IVL. High-resolution OCT identified the lesion as a rigid, circularly retracted segment caused by complete transverse fracture and elastic recoil, distinct from typical linear fracture patterns. Ultimately, lumen reconstruction was successfully achieved using a modified Side Flap Technique combined with a stent-in-stent strategy, thereby addressing an unmet clinical need for managing such mechanical complications following DES implantation. While traditional cardiovascular risk factors, including diabetes, hypertension, and dyslipidemia-are established contributors to neointimal hyperplasia and recurrent ISR, they do not fully account for the early, refractory, and mechanically resistant nature of the stenosis observed in this case. The angiographic and intracoronary imaging findings, coupled with the failure of high-pressure balloon dilation and IVL, indicate that a non-dilatable mechanical obstruction (specifically, the metal ring) was the dominant driver of recurrent ISR, rather than purely biological restenosis.

The underlying mechanism of stent fracture in this case is multifactorial. The mid-RCA exhibited severe S-shaped tortuosity with an angulation >45° and heavy calcification. Two long open-cell stents (each >25 mm) were implanted, leaving inter-strut gaps. High-pressure post-dilation precipitated strut fracture, and persistent mechanical stress from cardiac motion in the tortuous mid-RCA led to progressive metal fatigue and complete transverse fracture. The OCT-measured metal ring thickness (70 μm) matched the original stent strut thickness (75 μm), confirming the metal ring as a recoiled, fractured stent segment rather than a foreign body.

The mid-stent location and 70 μm thickness of the “metal ring” distinguish it from edge markers; complete strut interruption on longitudinal IVUS/OCT confirms stent fracture rather than accordion deformation; regular circular morphology and complete resistance to IVL exclude severe nodular calcification; and the intact withdrawal of the extension catheter, combined with the metallic signal characteristics on OCT, rules out foreign body retention. At the time of the third procedure, pre-intervention OCT revealed severe luminal restriction (minimum stent area 3.3 mm^2^ on prior IVUS) with a neointimal burden consistent with recurrent ISR. Although initial angiography after the index procedure suggested adequate expansion, subsequent imaging during recurrent ISR episodes confirmed early stent fracture with complete strut discontinuity.

Previous literature regarding refractory ISR etiologies has focused primarily on severe calcification or neoatherosclerosis (12, 13), which typically respond to excimer laser coronary atherectomy or IVL (14–16). However, the metal ring phenomenon in this case exhibits distinct pathological characteristics: unlike calcified nodules, it demonstrated remarkable resistance to IVL, suggesting mechanical strength far exceeding calcified plaques; the metal ring appeared as a continuous, regular ring-like high-density shadow unalterable by balloon dilation. Regarding the management of undilatable in-stent restenosis, rotational atherectomy (RA) theoretically offers a mechanism for metal reduction via stent ablation. In the present case, although pretreatment with IVL combined with high-pressure balloon dilatation achieved a lumen diameter of 2.08 mm at the site of the metal ring, the obstruction remained mechanically resistant and suboptimal. Given the distinct anatomical location of the metal ring and the procedural risks associated with the right coronary artery, we prioritized the modified Side Flap Technique combined with DES implantation to enhance procedural success rates while obviating the potential complications associated with RA, such as burr entrapment, vessel perforation, and slow flow. Nevertheless, for experienced centers, rotational atherectomy remains a viable salvage strategy within this therapeutic framework.

OCT appears to provide important diagnostic utility in the identification of such lesions. Therapeutically, the modified Side Flap Technique—employing a three-step approach of “stent strut crossing, ring displacement, and stent implantation”—displaced the metal ring against the vessel wall and restored the lumen. This strategy reduced procedural risks and averted the need for coronary artery bypass grafting due to recurrent ISR. Importantly, this technique advances the guidewire through the potential space between the fractured metal ring and the vessel wall, avoiding passage through the stent mesh and thereby eliminating the risk of creating another strut-related ring. Under OCT guidance, the new stent was implanted with optimized post-dilation, fixing the metal ring against the vessel wall and preventing further deformation. Thus, this approach appeared technically feasible in this case and may offer a potential salvage solution for managing such rare complications, though its reproducibility warrants validation in further cases.

## 4 Conclusion

In conclusion, this case confirms that for localized intractable ISR secondary to a non-expandable metal ring following DES implantation, pre-procedural OCT offers important diagnostic value in this context. Furthermore, combining the Modified Side Flap Technique with stent-in-stent implantation may serve as a feasible salvage revascularization strategy that achieved durable luminal patency in this patient, offering preliminary technical insights for coronary intervention.

## Data Availability

The data that support the findings of this study are available from the corresponding author upon reasonable request.

## Supplementary material

Supplementary Video 1 OCT pullback demonstrating luminal obstruction caused by the metal ring and restoration of patency with the ring apposed to the vessel wall following treatment.

Supplementary Video 2 CAG illustrating the key steps of guidewire passage lateral to the metal ring, balloon dilation displacing the ring to the vessel wall, and stent implantation.
